# Dr. J. J. Towle

**Published:** 1898-04

**Authors:** 


DR. J. J. TOWLE, of Jamestown, N. Y., died at his home in that city March 11, 1898, aged 61 years. He was seized with apoplexy and was found dead in his barn. He was a well-known physician and is survived by a widow, two sons and a daughter.

